# High-Throughput Approaches for the Identification of *Pseudomonas aeruginosa* Antivirulents

**DOI:** 10.1128/mBio.02240-20

**Published:** 2021-05-04

**Authors:** Donghoon Kang, Liyang Zhang, Natalia V. Kirienko

**Affiliations:** a Department of Biosciences, Rice University, Houston, Texas, USA; University of Texas Health Science Center at Houston

**Keywords:** antimicrobial resistance, antivirulence, high-throughput screen, type II secretion, type III secretion, biofilm, quorum sensing, drug discovery, antimicrobial resistance, *Caenorhabditis elegans*, *Pseudomonas aeruginosa*, biofilms, drug screens, pyoverdine, secretion systems

## Abstract

Antimicrobial resistance is a serious medical threat, particularly given the decreasing rate of discovery of new treatments. Although attempts to find new treatments continue, it has become clear that merely discovering new antimicrobials, even if they are new classes, will be insufficient. It is essential that new strategies be aggressively pursued. Toward that end, the search for treatments that can mitigate bacterial virulence and tilt the balance of host-pathogen interactions in favor of the host has become increasingly popular. In this review, we will discuss recent progress in this field, with a special focus on synthetic small molecule antivirulents that have been identified from high-throughput screens and on treatments that are effective against the opportunistic human pathogen Pseudomonas aeruginosa.

## INTRODUCTION

Pseudomonas aeruginosa is a particularly versatile bacterium, and its metabolic plasticity gives it a unique environmental ubiquity, including in health care settings. This makes the organism a clear and present threat to patients, particularly those who are immunocompromised, recovering from chemotherapy, organ transplantation, or serious burns, and those who have cystic fibrosis or other airway disorders (1, 2). Importantly, this same metabolic versatility gives rise to the ability of the pathogen to grow in a variety of host niches, causing a panoply of disorders, including sepsis, soft tissue, respiratory, and urinary tract infections, and keratitis. Unfortunately, the treatment of P. aeruginosa infection is complicated by the inherent resistance of the organism to a wide variety of antimicrobials due to low membrane permeability, expression of several families of efflux pumps that further limit treatment efficacy (3), and the production of biofilms that limit physical access of immune effectors and therapeutics (4, 5). Moreover, it readily acquires new resistance mechanisms via lateral gene transfer, which is likely the route that led to the emergence of carbapenemase-expressing strains of P. aeruginosa (6). These factors, combined with the growing threat of antimicrobial resistance in general, make it imperative not only that new antimicrobials be discovered but also that new types of treatments be developed.

**FIG 1:**
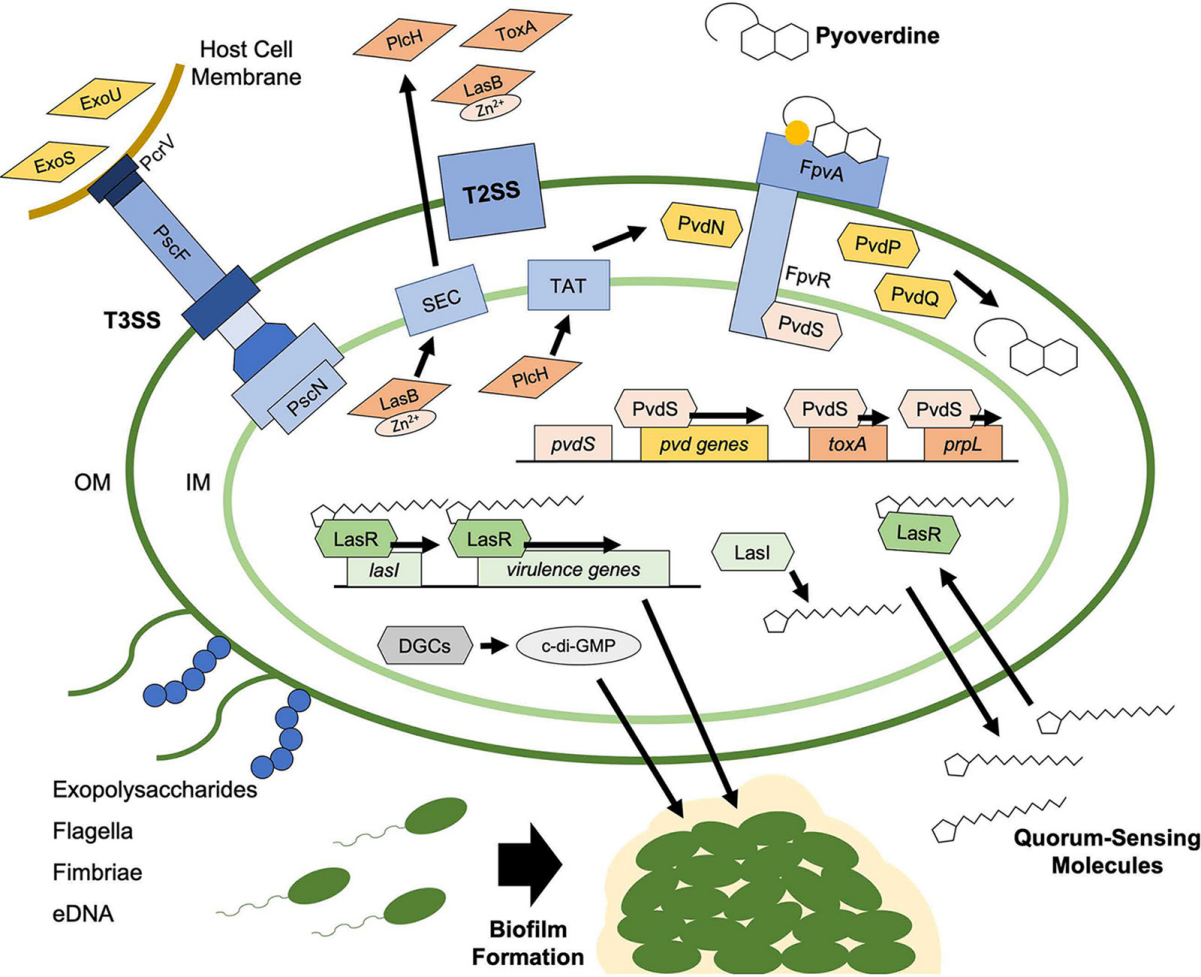
P. aeruginosa virulence factors for therapeutic intervention. Antivirulents identified from high-throughput screens inhibit quorum-sensing (QS), biofilm formation, toxin secretion (via T2SS or T3SS), or pyoverdine production or function. QS antivirulents inhibit autoinducers (e.g., acylhomoserine lactones and 2-alkyl-4-quinolones), their synthases (e.g., LasI, RhlI, and PqsABCDH), or their receptors (e.g., LasR, RhlR, and PqsR). Biofilm inhibitors often target regulators of formation, such as QS or c-di-GMP production, as well as components of the extracellular matrix. T2SS- and T3SS-dependent virulence targets have included effectors (e.g., LasB, PlcH, ExoS, or ExoU) and delivery systems like the twin arginine translocase (Tat) and the T3SS needle complex. Pyoverdine inhibitors have been found to hamper the production of pyoverdine and its function by binding to the siderophore directly.

One promising approach is to target the virulence determinants of the pathogen. Suppressing the ability of the bacterium to inflict damage is likely to reduce pathogenesis and facilitate bacterial clearance. Here, we will briefly review the methods for identifying antivirulents (summarized in Table 1) and discuss the most frequently targeted virulence systems. Generally, these approaches seek to block either biological signaling (which typically involves not only signal propagation but also signal amplification, making disruption more likely to be effective) or the pathogenic effectors themselves. Although virtually all virulence factors in P. aeruginosa have been the topic of therapeutic intervention, we will focus on three general targets that have been consistently validated in various mammalian infection models: quorum sensing (QS) and biofilm formation, the type II (T2SS) and type III (T3SS) secretion systems, and the siderophore pyoverdine (Fig. 1).

**TABLE 1:** Major high-throughput screening methods for the discovery of P. aeruginosa antivirulents

Methodology	Advantages	Disadvantages	Notable references
Transcriptional reporter	More amenable to high-throughput screening due to simple colorimetric or fluorometric outputs Reporter construct can be easily modified to screen for other virulence factors Highly versatile approach for bacterial factors involved in cell signaling (e.g., quorum sensing)	Difficult to identify biosynthetic inhibitors for virulence factors synthesized by multiple enzymes Requires understanding of virulence gene regulation	7 – 12
Enzymatic activity assay	Typically have simple readouts that can be measured using a plate reader Established substrates for various enzymes (e.g., protease, phospholipase, tyrosinase, lactonase)	Hits are limited to functional inhibitors Assay may not be specific due to functional redundancies in pathogen factors May require purification of drug target	13 – 16
Mammalian cell/whole-organism pathogenesis model	Can broadly screen for different classes of anti-infectives Screening conditions can be optimized (e.g., using various mutants) to target a specific subset of virulence factors Allows simultaneous counterscreening for cellular or organismal toxicity	Requires high-throughput methods to measure cell/organism survival Can be difficult to ascertain drug function if pathosystem is not fully characterized Bacterial drug absorption can be affected by host factors Substantially more costly than *in vitro* assays	17, 18
*in silico* screening	May reuse computational pipeline for various drug targets Allows for remote preliminary work Saves time and resources on developing assays and obtaining large diversity libraries Can allow for a much larger screen	Require extensive prior characterization of drug target (e.g., protein structure) for credible outcome and to reduce false-positive hit rate Less established method for drug discovery Can be computationally expensive and time consuming	19

## QUORUM SENSING AND BIOFILMS

In addition to limiting physical access to antimicrobials and immune effectors, the biofilms for which P. aeruginosa is well known also facilitate colonization of host tissue, medical implants, and environmental surfaces. A comprehensive discussion of the signals and mechanisms involved in biofilm formation is beyond the scope of this article, but several excellent reviews are available (20–22). In brief, the development of biofilm is a multistep process mediated by attachment using various motility factors followed by the secretion of extracellular matrix components, all of which is regulated by a well-characterized and hierarchical group of QS systems (23). Due to their importance, QS and biofilm are the most frequently targeted systems for disrupting P. aeruginosa virulence, and many chemical inhibitors have been identified.

### Quorum sensing.

The role of QS in virulence has been extensively studied and several well-written reviews are available (24–30). Briefly, P. aeruginosa expresses three main QS systems, *las*, *rhl*, and *pqs*, which produce the QS ligands 3-oxo-C_12_-homoserine lactones (C_12_-HSL), C_4_-homoserine lactones (C_4_-HSL), and *Pseudomonas* quinolone signal (PQS), which are secreted into the extracellular milieu. Higher bacterial density increases import of these molecules back into bacteria, where they act as signals of local bacterial population and trigger a variety of population-level growth behaviors (e.g., whether to grow in a planktonic or sessile lifestyle). In addition to regulating the production of biofilm, QS systems in P. aeruginosa also control expression of several other virulence factors, including elastase, alkaline protease, exotoxin A, and pyocyanin.

One of the first screens performed to identify QS inhibitors for P. aeruginosa utilized derivatized versions of its normal C_12_-HSL ligand, identifying several compounds that had varying ability to quench QS-dependent gene expression, including biofilms and virulence factor production (31). Another early effort involved a structure-based screen of known drugs and natural products and their predicted ability to interact with the ligand-binding domain of LasR, based on their similarity to known ligands (32). The compounds identified in this way reduced QS for all three systems, inhibited biofilm, and limited QS-dependent virulence. A more recent screen was performed to identify compounds with similarity to 3-methylene-1-tetradecylpyrrolidine-2,5-dione, which inhibits QS as well (33). After performing a structure-activity relationship (SAR) study, a pair of itaconimide derivatives with low micromolar efficacy for inhibiting QS was identified. Interestingly, one of the compounds seemed to function in both *lasR*-dependent and -independent fashions.

A more comprehensive early screen of a larger chemical space utilized a cell-based assay with a yellow fluorescent protein reporter driven by the *rsaL* promoter, which is activated by LasR (7). Using this approach, the authors were able to screen a diverse set of almost 200K compounds. Despite this, the two best hits were also very similar to C_12_-HSL. Like the previous hits, these hits reduced QS and QS-dependent virulence. Using a similar, transcription-based screen of a 16K member diversity library, another group found nine QS inhibitors (10). Interestingly, these hits were much more divergent in structure, which demonstrated the utility of a chemical diversity library, which should more efficiently explore chemical space (34). A pair of screens targeted PqsE, involved in the synthesis of PQS, using physical interaction techniques (35, 36). Although these screens also found several hits, they failed to inhibit virulence determinants. Ultimately, none of these screens were extended to investigate whether these hits could limit pathogenesis.

This concern was addressed in another high-throughput screen that utilized an elegant system where the *pqsA* promoter was used to drive expression of *sacB*, allowing almost 250K molecules to be screened in a simple live-dead format on sucrose-containing media (8). Using this approach, 17 compounds (from approximately five different scaffolds) were identified. The most promising was a group of at least eight compounds from a benzamide-benzimidazole scaffold. After completing an SAR study, the authors improved the efficacy of the compounds to block several QS-based phenomena, including signaling and virulence factor production, in the high nanomolar range. Importantly, they took the next critical steps, verifying that the compounds bound a key QS regulator, MvfR, that they prevent pathogenesis in two different murine virulence models, and that they work well in combination with antimicrobials. This study represents one of the best and most thorough characterizations of the potential of an antivirulent, and it will be exciting to see the development of these compounds.

Furthermore, several studies have also identified enzymes, such as lactonases or the P. aeruginosa homoserine lactone acylase PvdQ, that can chemically modify or degrade extracellular QS molecules, particularly C_12_-HSL and C_4_-HSL (37–39). These inhibitors are specifically referred to as quorum quenchers to distinguish them from other quorum-sensing inhibitors (QSIs). Various QSIs and quorum quenchers have been shown to attenuate the production of virulence factors *in vitro* and mitigate P. aeruginosa virulence in invertebrate and mammalian host models without exhibiting overt antimicrobial activity (8, 40–43). QSIs also increased bacterial susceptibility to antibiotics by inhibiting biofilm formation (8, 42, 44). To optimize anti-QS therapy, studies have also proposed strategies to treat P. aeruginosa with a combination of a QSI and a quorum quencher or two QSIs with distinct targets (i.e., QS molecule synthase and receptor), as these combinations synergistically mitigate the production of virulence factors (45, 46).

It is important to note that many P. aeruginosa QSIs were identified from the metabolites of other prokaryotic and eukaryotic organisms (47–51). For instance, E. coli produces indole and indole derivatives that mitigate QS-regulated virulence factors such as pyocyanin and rescue guinea pig hosts during P. aeruginosa lung infection (47). Similarly, halogenated furanones such as C-30, which inhibit P. aeruginosa QS *in vivo* and promote pathogen clearing in mouse lungs (42, 48), were derived from metabolites found in macroalgae. Based on the discovery of these natural products, there have been efforts to identify novel antivirulents from environmental microbes. Various studies screened bacterial environmental isolates (52), particularly *Streptomyces* strains (53, 54), for QS-inhibiting or quorum-quenching activity using similar biosensors as described above.

### Cyclic di-GMP signaling and biofilm formation.

Another approach to limit biofilm formation is to target it further downstream in the signaling and/or production process. Naturally, attempts at this have been made as well. Cyclic diguanylate (c-di-GMP) is a well-known signaling molecule in bacteria, controlling many aspects of biological activity, including the conversion to a sessile lifestyle and biofilm production, making it a potential target (55, 56). c-di-GMP is produced by a class of enzymes known as diguanylate cyclases (DGCs) (57).

The first screens to target c-di-GMP used enzymes from other bacterial species, with the assumption that DGCs would be largely conserved. One early experiment used an Escherichia coli strain that overexpressed a DGC called AdrA (58). The authors screened approximately 1,100 compounds and found a single hit, sulfathiazole, which has been used in the past as an antibiotic. Interestingly, the DGC inhibitory effect was independent of the antimicrobial activity. The Waters lab conducted a screen of approximately 66K compounds, using a c-di-GMP-induced reporter driving luciferase expression in Vibrio cholerae (59, 60). In the process, these authors identified 41 hits with half-maximal inhibitory concentration (IC_50_) values less than 1 μM. Characterization of the compounds demonstrated that they were effective at disrupting V. cholerae c-di-GMP production, but only a small handful worked on other DGCs. Of the seven compounds that did so, none were particularly effective against P. aeruginosa, with only one showing even moderate activity. Given that P. aeruginosa is predicted to encode approximately 40 DGCs (61), the only reasonable way to inactivate all of them would be to target the shared GG[D/E]EF motif that generates c-di-GMP, but saturating that site on dozens of enzymes seems impractical.

A more direct method of targeting biofilm has also been investigated. Most often, this type of screening leverages the fact that biofilms can be stained with crystal violet. The first attempt at this approach used ∼4.5K molecules and identified a single compound, ferric ammonium citrate, that limited biofilm growth (62). Careful analysis demonstrated that ferric iron was responsible and that increasing iron during growth would limit biofilm formation. Since hosts and pathogens are well known to compete for iron during infection, this route has not been given much further attention.

Another screen of flavonoids, oxazolidinones and plant extracts using crystal violet as a reporter found four hits, of which only palmitoyl-dl-carnitine was studied in depth (63). Palmitoyl carnitine was observed to inhibit LasI/LasR-mediated QS, but this was independent of its effect on biofilm. Interestingly, it was also independent of c-di-GMP levels and could override the biofilm stimulatory effects of aminoglycosides (63), a known outcome of subinhibitory dosing with this class of antimicrobials (64). Using a similar method, a group of hydrazine-carboxamide hybrid molecules were also found to inhibit biofilm production (65).

Other screens that have also been performed include a 66K compound screen using a luminescence-based assay that leverages ATP from lysed cells that had adhered to plates (66) and a physical interaction-based assay that measured c-di-GMP binding to PelD, an enzyme involved in the synthesis of the major exopolysaccharide, Pel (67). In the former screen, the authors identified 83 compounds, of which 30 had EC_50_ (the minimum concentration required to rescue 50% of the worms) values less than 20 μM, but no tests of the impact in an infection model were performed. The latter screen identified a single hit, ebselen, which covalently modifies cysteine residues in several DGCs, preventing their activity. The compound also repressed c-di-GMP-mediated changes in virulence.

As with QS inhibitors, there is a relative paucity of testing of DGC inhibitors *in vivo* or in infection models. One screen, using a *pelB*-driven luciferase reporter, involved ∼31K molecules and identified 14 compounds that repressed *pelB* expression without affecting growth. The compounds were able to prevent biofilm production, and several combinations with antimicrobials showed an additive effect. Importantly, four of the compounds also prolonged Caenorhabditis elegans survival during a biofilm-dependent infection (11).

### Other methods of targeting biofilm.

Other biofilm inhibiting compounds are also known to exist and have been reviewed elsewhere (68). For example, the Melander lab rationally designed several compounds (69) by linking the 2-aminoimidazole functional group found in the algal anti-biofilm metabolite bromoageliferin (70) with a menthyl carbamate moiety identified during the derivation of a marine bacterial exoproduct (71). The resulting compounds had efficacy in the midmicromolar range, although they were not effective at dispersing already-formed biofilms, suggesting that their activity prevents biofilm biosynthesis. Work from the Blackwell lab identified a group of 2-aminobenzimidazoles that could prevent P. aeruginosa biofilm formation or promote their dispersion after biofilms had already been established, including one compound that was effective at very low micromolar concentrations (72).

Another strategy is to promote biofilm dispersion. For example, it has been reported that varying the media and nutrient conditions can trigger this process (73, 74). Interestingly, targeting DGCs also appears to be useful here. An *in silico* approach identified several compounds that targeted the Caulobacter crescentus DGC PleD by screening ∼15K compounds, with a focus on commercially available products that resembled guanine/oroidin (75). Four compounds identified were effective at limiting DGC activity and successfully dispersed established biofilms, although relatively high concentrations (high micromolar) were required.

The Kelso lab took the approach of combining ceftazidime (a β-lactam) with a linker that releases two NO molecules, which have been shown to trigger biofilm dispersal, even at low concentrations (76), and a primary amine with weak antimicrobial activity as an “all-in-one” treatment molecule (77). These researchers screened a dozen derivatives of this general class of compounds (cephalosporin-3′-diazeniumdiolates). Their best hit showed activity against ceftazidime-resistant biofilms and was at least as effective as its parent molecule in a murine respiratory infection model.

Finally, some evidence suggests that combining theoretically “inert” compounds (called excipients) with antimicrobials can help improve treatment efficacy against biofilms. An early report on this phenomenon showed that mannitol, for instance, could improve tobramycin efficacy, probably by changing the osmolarity (78). Mannitol can also potentiate ciprofloxacin treatment (79). On this basis, Smyth and colleagues screened ∼200 excipients for the ability to improve tobramycin (80). These researchers found several hits, including l-alanine and succinate, that enhanced treatment.

The Waters lab followed up on this by screening ∼6.1K drugs with regulatory approval that were part of repurposing libraries (81). From this screen, the lab identified a small pool of drugs that were effective at synergizing with tobramycin but that had no significant antipseudomonal activity on their own. Among these was triclosan, often marketed as irgasan, which is a broad-spectrum antimicrobial that targets fatty acid biosynthesis (82, 83). From one perspective, this is surprising, since triclosan is well known to be ineffective against P. aeruginosa and it lacks any known antibiofilm properties (84). However, the Waters lab only removed *effective* antimicrobials from their pool of hits. Since this was done based on the ability of the hits to prevent growth, which triclosan did not do, it is logical that the hit would have remained in their pool. Further analysis demonstrated that triclosan synergized with other aminoglycosides, including gentamicin and streptomycin, and that this was effective against drug-resistant bacteria as well.

Surprisingly, although this effect was specific to P. aeruginosa in biofilms (no synergy was observed in planktonic bacteria), the combination did not seem to increase biofilm dispersion (81). Instead, the compound appears to disrupt the proton-motive force that is used to power RND pump-mediated drug efflux, increasing intracellular tobramycin concentrations (85). This result remains somewhat intriguing, since efflux is generally accepted as the mechanism of P. aeruginosa resistance to triclosan (86, 87). It is also unclear why this would be specifically effective in biofilms. It may suggest that some aspect of a sessile lifestyle renders them more susceptible to disruptions of the proton motive force. Given the importance of this system in the well-known efflux of otherwise effective treatments, this is an intriguing target for future study.

## TOXIN SECRETION SYSTEMS

Most Gram-negative bacteria encode several toxin secretion systems. P. aeruginosa produces at least five distinct systems that use different components and structures and even use different methods for translocating materials (88). These systems deliver various virulence factors and toxins either into the extracellular milieu or directly into host cells. Several of these systems have been targeted for the development of antivirulents, but only the type II and type III secretion systems will be focused on in this review. A comprehensive recent review covered additional systems and drug discovery methods that are not discussed here (89).

### Type II secretion system.

The type II secretion system (T2SS) utilizes a two-step transfer, wherein the toxins are first transported to the periplasm of the bacterium via the Sec or twin arginine translocase (Tat) complexes and then exported across the outer membrane via a pair of different T2SS systems (88). Toxins secreted by the T2SS include exotoxin A, which inhibits translation by ADP-ribosylating elongation factor-2 (90), hemolytic phospholipase C, which cleaves cellular lipids and causes lysis (91), and the elastase LasB, which degrades a wide variety of substrates, including immune proteins and antimicrobial peptides (92). LasB also aggravates epithelial injury by cleaving cadherins, disrupting cell-to-cell junctions (93).

Several screens have been performed to identify inhibitors of Tat. The first screen used ∼75K compounds to search for hits that prevented the activity of a *PA1365*::*lux* reporter, and resulted in the discovery of ∼50 hits (94). The compounds blocked the secretion of both LasB and the phospholipases PlcH and PlcN. Another screen leveraged a chromogenic substrate to assay ∼80K compounds for the ability to prevent secretion of PlcH (14). A total of 122 primary hits were found, but secondary assays for the secretion of pyoverdine maturation factors (see below), copper resistance, and facilitated growth on choline as the sole course of carbon and nitrogen, all of which are associated with Tat activity, limited this number to ∼40 compounds. Eventually, two compounds emerged as the most promising candidates, although Forsberg and colleagues would later argue that they were still cytotoxic, immunomodulatory, and unlikely to be appropriate for further development (15). In a more recent screen, they used a similar chromogenic PlcH substrate to independently assay ∼39K compounds (15). This effort led to the identification of five Tat inhibitors and one T2SS inhibitor that was similar to those discovered by Moir et al. (94).

Inhibitors that directly target LasB have also been identified. Using a combination of *in silico* docking and *in vitro* testing, Zhu et al. identified a mercaptoacetamide-based thiol scaffold to develop a compound that effectively limited LasB-dependent virulence in C. elegans without impacting other matrix metalloproteases (19). Another group, using a solely *in vitro* screening approach, assayed ∼1.5K small molecules and identified a single mercaptoacetamide-based thiol that inhibited LasB activity and rescued Galleria mellonella from P. aeruginosa pathogenesis (95). A careful analysis demonstrated that the compound interacted with the zinc-based active site of the metalloprotease, which is likely to be the mechanism for all mercaptoacetamide-based LasB inhibitors. Subsequent research refined this compound and identified a more effective analog (96). The refined inhibitor exhibited minimal activity toward human matrix metalloproteases and little cytotoxicity.

### Type III secretion system.

Unlike some of the other pathogenic determinants described here, the presence and activity of the type III secretion system (T3SS) is often dispensable for infection, although it does contribute to the virulence of the pathogen in a variety of animal models and in human disease (97). Mechanistically, the T3SS delivery system is comprised of nearly a dozen proteins that form an ultrastructure reminiscent of the form and function of a hypodermic needle built from two protein assemblies that give rise to the “needle” translocation complexes. In P. aeruginosa, the T3SS delivers at least seven proteins into the cytoplasm of host cells, including four well-known toxins: ExoS, ExoT, ExoU, and ExoY (97, 98). ExoS and ExoT are bifunctional GTPase-activating and ADP-ribosylating proteins that trigger apoptosis. ExoU is a phospholipase that causes rapid eukaryotic cell lysis. ExoY is a nucleotidyl cyclase that seems to stabilize actin filaments, limit phagocytosis, and disrupts cell junctions (99, 100). Although most strains of P. aeruginosa only carry either ExoS or ExoU, the strains that carry both tend to exhibit high-risk, multidrug resistance phenotypes (101, 102). The T3SS can also trigger pathogenesis independently of effector delivery by activating the NLRC4/IPAF inflammasome, causing pyroptosis (103).

As with T2SS, the delivery system for T3SS has been targeted. Aiello and colleagues used a luciferase reporter driven by the *exoT* promoter to screen for changes in secretion and identified approximately five scaffolds that were effective (of 80K compounds screened) (12). These researchers discovered that phenoxyacetamides, malic diamides, and *N*-phenylmaleimides could limit *exoT* gene expression. Further counterscreening led to the identification of a single promising hit, MBX 1641, that inhibited the T3SS of P. aeruginosa, Yersinia pestis, and Chlamydia trachomatis. Further study of this compound demonstrated that it acts on PscF, the needle protein of the T3SS (104).

Two more recent studies also targeted the needle complex. The first utilized a physical-based assay to determine whether small molecules limited the formation of the PscEFG heterotrimer, which prevents premature aggregation of PscF monomers (105). In a serendipitous turn of events, tashinones—a family of plant-derived compounds expected to serve as a negative control for assay development—exhibited strong binding, preventing the formation of the T3SS system. In this impressive study, the authors demonstrated that several compounds from this family of molecules limited pathogenesis in both murine macrophage infection and murine pneumonia models. Importantly, tashinones have this effect even if treatment occurred after pathogen infection. The authors determined that the effect of tashinones appears to use a different phenomenon than the aforementioned MBX 1641, which they also showed limits secretion and reduces macrophage cytotoxicity.

Finally, a different group used a clever set of ELISAs featuring PscE and PscG as “bait” and signal (106). Compounds that bound to the bait protein and prevented the formation of the heterodimer were then combined using synthetic chemistry approaches to develop larger, more complex molecules with increased affinity and efficacy, an approach that is increasingly commonly used in the development pipeline for drugs coming from fragment-based screening (34). The screen identified several fragments that bound to each of the proteins, allowing the group to investigate a number of permutations. Several were very effective *in vitro*; two prevented T3SS secretion and one partially limited pathogenesis in a G. mellonella model.

T3SS-mediated pathology can also be reduced by targeting individual toxins (107). Since most T3SS-mediated toxins directly cause host cell death, several studies have utilized cytotoxicity assays to identify chemical inhibitors. For instance, a high-throughput screen for ExoS inhibitors was performed by expressing ExoS under an inducible promoter in Saccharomyces cerevisiae (108). A screen of 56K compounds identified six potential hits, one of which, named exosin, limited ExoS-mediated killing of Chinese hamster ovary (CHO) cells.

Another frequent approach has been to screen for compounds that could prevent the delivery of the cytotoxic effectors. The earliest such assay involved screening 50K compounds for their ability to rescue CHO cells from infection with P. aeruginosa (18). Of the 88 hit compounds, one, named pseudolipasin A, appeared to specifically prevent the activity of ExoU and was effective at preventing pathogenesis in a Dictyostelium discoideum model. However, further attempts to optimize the compound met with limited success (109). A similar approach, using A549 airway epithelial cells to screen 10K compounds identified a single scaffold that showed some potential to inhibit both QS and T3SS function (110), although the screening concentrations were fairly high. Interestingly, this scaffold had also been identified previously in a targeted fashion as a biofilm inhibitor (47).

## PYOVERDINE

One critical virulence determinant during acute infections with P. aeruginosa is the siderophore pyoverdine. Siderophores are small molecules secreted by pathogens to aid in the acquisition of ferric iron, which can otherwise be difficult to obtain in aqueous environments. They also contribute to virulence by providing iron during infection by removing this transition metal from iron-sequestering proteins, a process that hosts assiduously try to prevent (111).

Pyoverdine contributes to host pathogenesis in several key ways. First, uptake of iron-bound pyoverdine releases the sequestration of an alternative sigma factor, PvdS, that regulates the expression of several P. aeruginosa toxins, including exotoxin A, the protease PrpL, and the production machinery for pyoverdine itself (112, 113). Pyoverdine production (114), and more broadly iron homeostasis (115), also supports P. aeruginosa biofilm formation. Finally, pyoverdine itself functions as a toxic product, entering C. elegans tissue or mammalian macrophages, removing iron and causing damage to host mitochondria (116–119). Some combination of these activities drive the oft-observed requirement for pyoverdine for pathogenesis in invertebrates and mice (120–124).

Pyoverdine biosynthesis and export are a complicated process mediated by at least 14 biosynthetic enzymes, as the siderophore is comprised of both an invariant core and an oligopeptide sidechain (113, 125). Biosynthesis is currently thought to occur in so-called “siderosomes” that exist at the interface between the cytoplasm (where the peptide moiety is assembled) and the periplasm (where the fluorescent core is manufactured) (126). As noted above, the twin arginine translocase (Tat) is required for the periplasmic transport of PvdN (127) and PvdP (128), making the Tat inhibitors described above potential inhibitors of pyoverdine as well, and attenuate pyoverdine-mediated virulence (14).

Perhaps unsurprisingly, a variety of high-throughput screens that targeted pyoverdine biosynthesis have been performed. Notably, small molecules that directly bind to and inhibit the tyrosinase PvdP and acyl-homoserine lactone acylase PvdQ have been identified. Each of these proteins exhibit distinct enzymatic functions beyond their role in pyoverdine maturation, which have facilitated high-throughput biochemical screening. For instance, inhibitors of PvdP were identified from known tyrosinase inhibitors by measuring the *in vitro* conversion of l-tyrosine to dopachrome by the enzyme (129). One of the earliest screens targeted the maturation factor PvdQ and used fluorogenic or chromogenic substrates to screen ∼1,300 U.S. Food and Drug Administration (FDA)-approved drugs (the LOPAC collection) and identified two potential hits (130). Using the same approach, a larger library of compounds (∼340K) was screened for additional PvdQ inhibitors and 89 compounds with IC_50_ values of <10 μM were found (16, 131). However, this efficacy was limited in strains actively effluxing drugs via MexAB-OprM. Nevertheless, even in these strains, their best hit, a biarylnitrile, inhibited pyoverdine production at physiologically relevant concentrations (16).

As with many systems, targeting signaling has been attempted with pyoverdine. This makes intuitive sense, as most signals undergo amplification, making it easiest to stop them as early as possible. Since PvdS drives expression of multiple pyoverdine biosynthesis genes, it is a rational place to start. In addition, PvdS shows greater sequence conservation across pseudomonads than many of the enzymes involved in pyoverdine maturation (132). To identify inhibitors of PvdS, Imperi and colleagues used the *pvdE* promoter (which depends on PvdS) to drive luciferase expression and screened FDA-approved drugs under iron-limiting conditions (9). They identified a single hit, 5-fluorocytosine (5-FC). Careful experiments demonstrated that effect from 5-FC required its conversion to the well-known cancer chemotherapeutic 5-fluorouracil (5-FU), and that 5-FC mitigated both pyoverdine-dependent virulence and P. aeruginosa pathogenesis in a murine infection model.

Around this same time, we serendipitously identified 5-FU in a high-throughput screen for pyoverdine-dependent pathogenesis using a C. elegans platform (17, 123). Like Imperi et al., we were able to determine that 5-FC mitigated virulence, including in mice (124), but we discovered that both compounds were metabolized to 5-fluorouridine and that the latter compound was directly responsible for disrupting pyoverdine biosynthesis, although the mechanism remains unclear. Although 5-FC is very effective as a treatment and can be efficiently delivered as an inhalable dry powder (133), the development of resistance is surprisingly rapid (see below), limiting its use.

In the previously mentioned screen, we leveraged C. elegans as a platform for the identification of novel antivirulents to limit P. aeruginosa killing. The assay used death as the readout for a screen of ∼86K compounds. This methodology has several advantages, including the reduction of false hits that are either toxic to the host or that will be successful *in vitro* only to be pumped back out of the pathogen, as has been the case for several other antivirulents. Of the ∼100 hits identified, four appeared to function (at least partially) by binding pyoverdine. Two of these also exhibited antimicrobial activity. The other two compounds, called LK11 and LK31, showed the ability to mitigate pathogenesis in C. elegans and synergize with clinically relevant antimicrobials making them promising candidates for further development (134).

Pyoverdine has one additional useful characteristic: the invariant dihydroxyquinoline core that provides the catecholate moiety, comprising two of the chelation sites, is also intensely fluorescent, making it trivial to identify pyoverdine in solution. Importantly, this fluorescence is quenched when the siderophore binds iron. Interestingly, the four pyoverdine inhibitors we previously identified also quenched fluorescence. A high-throughput biochemical screen of ∼45K compounds for hits that would quench pyoverdine fluorescence as a proxy of preventing virulence resulted in the identification of two compounds, each of which limited pathogenesis in the C. elegans model (135). A commercially available analog of one compound, named PQ3c, physically interacted with the core of pyoverdine, near its iron-binding site (135). Interestingly, the compound was effective despite having an affinity for pyoverdine (*K_d_* = 2.67 μM) thousands of times lower than that of pyoverdine for ferric iron (10^32^ M^−1^). Our current hypothesis is that Fe^3+^ chelation by pyoverdine occurs in a stepwise process, with the initial steps having a lower affinity than the final complex. If this is the case, it may be possible to disrupt pyoverdine-iron complex formation without requiring compounds to match the staggeringly high affinity of the siderophore for iron.

Finally, the regulatory relationships between pyoverdine and other virulence determinants may facilitate the development of highly versatile antivirulents. For example, we found that antibiofilm agents can also inhibit pyoverdine production (136). The likeliest explanation for this was that bacterial aggregation was a necessary prerequisite for the initiation of pyoverdine biosynthesis (137, 138). In addition, several QS inhibitors have been reported to attenuate pyoverdine production (139–142), including 5-FU, which was identified as an inhibitor of QS in P. aeruginosa before it was known to disrupt pyoverdine biosynthesis (143).

## ARE ANTIVIRULENTS THE FUTURE?

Despite almost 20 years of research and a steady increase in interest, no antivirulents have yet been introduced for clinical use. At least part of this can be attributed to the general disinterest of pharmaceutical companies to investigate this space. It is a well-observed fact that drug companies have little interest in the development and marketing of treatments for self-resolving conditions, since the cost of development of new antimicrobials will almost certainly never be recouped the way treatments for chronic conditions are (144). Frustratingly, the market for antivirulents is likely to be even worse, since these compounds face some of the same challenges as conventional antimicrobials and new obstacles as well.

For example, bacteria can develop resistance to antivirulents as well, although the pressure to do so is generally assumed to be lower. Generally, antivirulents can be broken into two categories: pure virulence inhibitors (i.e., those that prevent pathogenic determinants without affecting growth) and dual-function compounds that both reduce virulence and also limit bacterial growth. The second group is likely to be far more common, as selective pressures in bacteria (especially in hosts) are likely to stringently limit the expression of genes that are not directly necessary for survival. In either case, the development of resistance is a possibility, although the likelihood seems less with the former class. Indeed, studies have already revealed the development of single-gene mutation causing resistance to gallium and 5-fluorocytosine, two of the earliest antivirulence compounds identified (145–147). It remains to be seen how generalizable the phenomenon of antivirulent resistance is.

Additionally, there is overwhelming evidence that the expression of virulence effectors during P. aeruginosa infection is heterogeneous. For example, ExoU expression is a key difference between PA14 and PAO1, two commonly used laboratory strains, and varies in other clinical and environmental isolates as well (148, 149). The expression of pyoverdine, often recognized as critical for bacterial survival and growth *in vivo*, is even more well-known for its variability, especially in cystic fibrosis patients. We recently reported a wide range of pyoverdine production from isolates obtained from pediatric cystic fibrosis patients (124). Several well-known studies have demonstrated the accumulation of mutations that would preclude pyoverdine biosynthesis, suggesting pyoverdine may be dispensable late in chronic infections (150–153). In addition, there is clear evidence of a shift in iron uptake strategies away from siderophores toward heme-mediated ferrous iron acquisition (152, 154, 155).

Chronic bacterial infections tend to exhibit marked genetic heterogeneity (as demonstrated by changes in their nutrient utilization). Siderophores are a secreted product and are available for whatever bacterial cells express an appropriate receptor. This has two predictable consequences. First, evolutionary pressure to stop producing pyoverdine should increase, at least as long as the benefit to “cheating” (i.e., using pyoverdine produced by another cell) outweighs the cost of lower iron availability. Second, a disconnect between pyoverdine biosynthesis and pyoverdine receptor expression is likely, since the former is less favorable than the latter. It would be interesting to see a longitudinal study comparing expression of these genes, which may provide a more definitive answer to this long-standing question. Unfortunately, we are unaware of any such study.

Heterogeneity has also been observed in QS. For example, *lasR* mutants are occasionally observed in chronic infections (153, 156, 157). This is surprising, as LasR is often considered critical for the expression of other QS regulators, including RhlR (158, 159). However, *lasR* mutants partially retain their virulence due to residual expression of RhlR and functional redundancies between the transcription factors (160–162). For example, recent longitudinal studies showed that *lasR* mutants can activate the RhlIR QS system independently when elastases are required for growth (163, 164). Like other virulence determinants, the efficacy of targeting QS remains an open question.

Finally, it is hard to imagine antivirulents being clinically used as monotherapies. Instead, it is likely that they would be used in combination with antimicrobials, much as β-lactamase inhibitors like clavulanic acid are increasingly used with β-lactams. Several studies have reported promising synergy between antimicrobials and antivirulents. Many of these directly or indirectly target P. aeruginosa biofilms to increase bacterial susceptibility to antimicrobials (13, 42, 44, 165–170).

## CONCLUSIONS

To date, no antivirulents have been approved for clinical use. Indeed, it is not clear that any have even proceeded to clinical trials. These treatments are often met with skepticism, and there is some basis for this opinion. Even the strongest antivirulents will, by necessity, be less effective than a new antimicrobial. However, it is becoming increasingly clear that the choice between antivirulents and antimicrobials will eventually disappear, as the latter drugs will become obsolete. Given the threat posed, it is essential that the development of these treatments, and their transition to clinical use, must be accelerated.
